# Cardiovascular Health and Cognitive Function: The Maine-Syracuse Longitudinal Study

**DOI:** 10.1371/journal.pone.0089317

**Published:** 2014-03-03

**Authors:** Georgina E. Crichton, Merrill F. Elias, Adam Davey, Ala'a Alkerwi

**Affiliations:** 1 Nutritional Physiology Research Centre, University of South Australia, Adelaide, Australia; 2 Centre de Recherche Public Santé, Centre d'Etudes en Santé, Strassen, Grand-Duchy of Luxembourg; 3 Department of Psychology, University of Maine, Orono, Maine, United States of America; 4 Graduate School of Biomedical Science and Engineering, University of Maine, Orono, Maine, United States of America; 5 Department of Public Health, Temple University, Philadelphia, PA, United States of America; University Medical Center Rotterdam, Netherlands

## Abstract

**Background:**

Smoking, physical inactivity, and poor diet, along with obesity, fasting glucose and blood pressure have been independently associated with poorer cognitive performance. Few studies have related scales representing a combination of these variables to multiple domains of cognitive performance. The aim of this study was to investigate the association between overall cardiovascular health, incorporating seven components, and cognitive function.

**Methods:**

A cross-sectional analysis employing 972 participants, from the Maine-Syracuse Longitudinal Study was undertaken. Four health behaviors (body mass index, physical activity, diet, smoking) and three health factors (total cholesterol, blood pressure, and fasting plasma glucose) were measured. Each was categorized according to the American Heart Association definitions for ideal cardiovascular health, except diet, for which two food scores were calculated. A Cardiovascular Health Score was determined by summing the number of cardiovascular metrics at ideal levels. Cognitive function was assessed using a thorough neuropsychological test battery.

**Results:**

Cardiovascular Health Score was positively associated with seven out of eight measures of cognitive function, with adjustment for age, education, and gender. With further adjustment for cardiovascular and psychological variables, these associations remained significant for Visual-Spatial Memory, Working Memory, Scanning and Tracking, Executive Function and the Global Composite score (*p*<0.05 for all). Ideal levels of a number of health factors and behaviors were positively associated with global cognitive performance.

**Conclusion:**

Increasing cardiovascular health, indexed by a higher number of metrics at ideal levels, is associated with greater cognitive performance. Smoking, physical activity, and diet are important components of cardiovascular health that impact upon cognition.

## Introduction

Modifiable risk factors are important in the management of cardiovascular disease (CVD) and cognitive functioning. Smoking, physical inactivity, and poor diet, along with obesity, hypertension and elevated blood glucose levels have been independently associated with poorer cognitive performance and dementia in later life [1]–[7]. Many models have been developed for the prediction of diagnosis and prognosis of CVD, typically comprising traditional risk factors including age, sex, hypertension, diabetes mellitus, smoking and blood cholesterol concentrations [8]–[11]. Only a scarce number of studies have used a CVD risk factor scale and related it to cognitive functioning. This has been done using the Framingham risk scores (Stroke Risk Profile and Cardiovascular Disease Risk Profile [12], [13]), risk factor scales that predict the incidence of cardiovascular events [14], [15]. However like most risk prediction models, these scales do not include positive health behaviors such as diet or physical activity [16]. One risk score that we are aware of, developed for the prediction of dementia from the population-based Cardiovascular Risk Factors, Aging, and Dementia (CAIDE) study, does include physical activity [17]. As these health factors often co-exist with traditional CVD risk factors and may share similar physiological pathways [18], [19], it is important to examine all these risk factors together in relation to cognition. Improvements in the cardiovascular health profile of individuals may help to prevent or slow age-related cognitive decline or dementia.

The American Heart Association (AHA), in its recently released ‘Strategic Impact Goal Through 2020 and Beyond’ defined ideal levels for four health behaviors (non-smoking, physical activity, diet, and appropriate weight as indicated by body mass index (BMI)), and three health factors (total cholesterol, blood pressure (BP), and fasting blood glucose), to identify ideal cardiovascular health [20]. This concept was described to promote substantial CVD reduction by 2020. Subequently, the number of ideal cardiovascular health metrics has been shown to be a strong predictor of mortality from all causes [21]. These findings, along with the work of others, has provided support for cardiovascular health as a valid construct [22]. To date, the Coronary Artery Risk Development in Young Adults study (CARDIA) is the sole longitudinal study that has examined associations between ideal cardiovascular health and cognitive function [23], showing that ideal cardiovascular health in young adulthood is associated with improved psychomotor speed, executive function and verbal memory in midlife.

The AHA has highlighted the importance of targeting cardiovascular health in order to maintain or improve brain health [24]. World-wide, 35.6 million people were estimated to be living with dementia in 2010, with current costs estimated at US$604 billion per year [25]. These costs are predicted to increase even more quickly than the future increase of prevalence.

The objective of the present study was to investigate the combined association of these seven cardiovascular health metrics with cognitive performance in a community-based sample of American adults. The present study is intended to extend existing findings by measuring five cognitive domains (indexed by multiple cognitive tests), and two global measures of cognition. Additionally we utilise a more detailed dietary metric than that defined by the AHA to better reflect adherence to current national dietary recommendations. It was hypothesised that a higher Cardiovascular Health Score (CHS; indicating a greater number of ideal health metrics) would be associated with better cognitive performance. Furthermore, each metric included in the CHS would be related to the overall global composite of cognitive scores.

## Methods

### Ethical Statement

The MSLS was approved by the University of Maine Institutional Review Board. All participants provided informed written consent for data collection and medical record review.

### Participants

Data were obtained from wave 6 of the Maine-Syracuse Longitudinal Study (MSLS) [26], [27]. At wave 1 MSLS participants were recruited for studies of blood pressure and cognitive performance with no recruitment exclusions other than institutionalization, diagnosed psychiatric disorder and alcoholism. Participants for the present study were those returning for the sixth (2001–2006) study wave as dietary intake measures were first obtained at this examination. Details of initial study recruitment have been previously described [26]–[29]. Beginning with 1049 individuals, participants were excluded for the following reasons: missing dietary or cognitive data (*n* = 34), acute stroke (*n* = 28), probable dementia (*n* = 8), undertaking dialysis treatment (*n* = 5), inability to read English (*n* = 1), and prior alcohol abuse (*n* = 1). Acute stroke was defined as a focal neurological deficit persisting for >24 hours and probable dementia was defined by cognitive measures, medical records and a multidisciplinary dementia review using the National Institute of Neurological Diseases and Communicative Diseases and Stroke/Alzheimer's Disease and Related Disorders (NINCDS-ADRDA) criteria [30]. Dementia cases were excluded as we were interested in examining relationships between cardiovascular health and cognitive performance, but not in those with severe cognitive impairment. The final sample with complete wave 6 data comprised 972 individuals, aged 23 to 98 years.

### Procedure

#### Health factors

Standardised protocols for data collection were used at all study visits. A blood sample was obtained following fast from midnight. Standard assay methods were employed [26] to obtain total cholesterol and fasting plasma glucose. BP was obtained after a light breakfast. Automated blood pressure measures (GE DINAMAP 100DPC-120XEN, GE Healthcare) were taken five times each in reclining, sitting and standing after a supine rest for 15 minutes, and averaged for systolic blood pressure (SBP) and diastolic blood pressure (DBP).

#### Health behaviours

Body weight was measured with participants wearing light clothing to the nearest 0.1 kg, and height was measured with a vertical ruler to the nearest 0.1 cm. BMI was calculated as weight in kilograms divided by height in metres squared. Physical activity was measured with the Nurses' Health Study Activity Questionnaire, a validated measure of time spent engaging in various physical activities [31]. MET-hours per week for each activity were calculated and summed to give total MET-minutes per week [32]. Smoking status (never, former, current) was based on self-report from the Nutrition and Health Questionnaire [33].

Dietary intake was assessed using the Nutrition and Health Questionnaire [33]. Participants are required to stipulate how frequently they consume a list of foods, with six response options (never, seldom, 1 time per week, 2–4 times per week, 5–6 times per week, ≥1 time per day). Portion or serving sizes are not stipulated; the totals are an estimate of intake in terms of times per day. For the diet metric, a Recommended Food Score (RFS) [34] and a non-Recommended Food Score (non-RFS) [35] were calculated, which differs from the healthy diet metric defined by the AHA. The RFS included 23 items: four types of fruit, eight types of vegetables and legumes, five types of wholegrain cereal products, two types of fish, two low fat milk products, nuts and seeds, and olive oil. These items are similar to those used previously [34], [36]. Consumption of any of the recommended foods at least once per week was assigned 1 point, otherwise 0 points if consumed less often [34]. A total RFS out of 23 was calculated, with a high score indicating a higher consumption of recommended food items.

The non-RFS [35] was calculated as the second aspect of the diet metric and included 15 items: processed meats, four whole-fat dairy products, two types of refined grains, solid fats/added sugars group, and alcohol. A score of 1 was assigned for consumption of non-recommended foods at least 2 to 4 times per week; otherwise 0 points were assigned if consumed less often than this [36], [37]. A total non-RFS out of 15 was calculated, with a higher value indicating a higher consumption of non-recommended food items.

#### Cardiovascular Health Score

Poor, intermediate and ideal levels for smoking, BMI, physical activity, total cholesterol, blood pressure, and fasting plasma were determined, based on the AHA definitions [20]. For each component, participants were given a score of 1 if they met the ideal AHA criterion, otherwise 0 points were assigned. Concerning diet, the highest 15% of RFS scores (≥14/23) and the lowest 15% of the non-RFS scores (≤1/15) were given a score of 1. Definitions of poor, intermediate and ideal health for each cardiovascular health component, and the prevalence of each in the present study can be found in Table S1. A total CHS was calculated ranging from 0 (no cardiovascular health metric at ideal levels) to 8 (all cardiovascular health metrics at ideal levels), and then categorised into low (0–2 metrics at ideal levels), medium (3–4) or high (5–8) cardiovascular health.

The metrics have not been weighted in the construction of the CHS as they have been used with equal weights in previous studies that afford important comparisons with the present study. It has been established that equal weighting results in scales that are more robust across studies [38].

#### Cognitive Function

The MSLS neuropsychological test battery comprises 20 individual tests designed to measure a wide range of cognitive abilities, and has been used in multiple studies examining cardiovascular risk factors and cognitive performance [26]–[28]. Prior to this study, these tests were used to derive five composite scores via factor analysis: Visual-Spatial Memory and Organization, Scanning and Tracking, Verbal Episodic Memory, Executive Function, and Working Memory [26]. The WAIS Similarities Test [39], a measure of abstract reasoning, loaded on all composite scores (factors) and was thus employed separately. The tests used to define each composite and the factor analytic methods used to derive these composites have been described previously [26]. A Global Cognition Composite score was derived by averaging the *z*-scores for all individual tests. In addition, the MMSE [40], a global measure of mental status widely used in the literature, is included in the MSLS battery. All cognitive performance measures are expressed in the same unit of measurement (SD units).

#### Covariates

Potential confounding factors were identified on the basis of two criteria: (1) had to be theoretically relevant [41] and, (2) had to show a statistically significant association (*p*<0.05) with both the CHS and the Global Composite. Covariates employed in the regression models (described below) were age (years), education (years), gender, total serves per day of all foods, depression (Center for Epidemiological Studies Depression Scale, CES-D, raw score), plasma homocysteine (tHcy, µmol/l), high sensitivity C-reactive protein (hs-CRP, mg/l), and triglycerides (mg/dl). Education level was obtained through self-report and ranged from 4 to 20 years. Total serves per day of all foods was estimated by summing the total daily intake of all foods included in the Nutrition and Health Questionnaire. The CES-D [42] was used to assess depression and the raw score used as a continuous variable. Plasma tHcy, triglycerides and hs-CRP were determined from blood samples using standardised MSLS blood sampling techniques [26].

### Statistical Analysis

Initially, descriptive analyses were performed to compare the demographic, health, and lifestyle characteristics of participants according to their cardiovascular health using the groups defined above (low, medium and high).

For the primary analyses, general linear modelling was used to examine the association between cardiovascular health, indexed by the CHS (the independent variable), and cognitive function (dependent variable). The CHS was employed as a categorical variable. Potential confounding factors were identified as described above and the following covariate sets were employed:

1. Basic covariate set: age, education, gender
2. Full covariate set: Basic + total serves per day all foods, CES-D score, hs-CRP, tHcy, triglycerides.

Analyses of linear and quadratic trends were performed, and polynomial trend analyses were conducted across the three levels of cardiovascular health. Adjustments for multiple comparisons were made and reported in terms of the Bonferroni adjustment. To confirm our findings, alternative analyses were subsequently performed using multivariate linear regression, utilising the CHS as a continuous variable (see Table S2).

Associations between each of the eight individual health behaviors and factors (defined as poor, intermediate, or ideal according to the AHA) and cognitive function (Global Composite) were then examined using general linear modelling. The Global Composite score was selected as the cognitive outcome because it represents the sum of a wide range of cognitive abilities. Two covariate sets were employed:

1. Basic covariate set: age, education, gender
2. Full covariate set: Basic + seven other cardiovascular health components. For example, when assessing BMI and the Global Composite, the seven remaining health components (smoking, physical activity, RFS, non-RFS, total cholesterol, BP, and fasting blood glucose) were included in the model. Multiple group comparisons were adjusted using the Bonferroni method. The data were plotted as figures. All analyses were performed using SPSS (version 20; SPSS, Chicago, IL).

Preliminary analyses indicated non-significant interactions beween age (in years) and the CHS (*p*>0.06) and between gender and CHS (*p*>0.20). Consequently these terms were not included in the covariate sets described above. Multicollinearity in the two covariate sets was examined using the variance inflation factor coefficient and was found to be within acceptable limits (VIF<1.2) (data not shown).

## Results

### Sample Characteristics

Table 1 shows the demographic, health and lifestyle factors of the MSLS participants according to cardiovascular health. Nearly one-half of participants (48.8%) achieved ideal health on 3 to 4 health metrics. Five or more metrics at ideal levels were achieved by 13.8% of the sample. Those who met a greater number had a higher number of education years, fewer depressive symptoms, and were less likely to be on medications (all *p*<0.05). They had significantly lower levels of tHcy, triglycerides, hs-CRP, and smaller waist circumference (all *p*<0.01). Mean levels of the health behaviors and factors were significantly associated with the total number of ideal health metrics as expected.

**Table 1:** Demographic, health and lifestyle factors of the MSLS sample (*N* = 972) according to Cardiovascular Health Score.

	Cardiovascular Health Score (0–8)						
Characteristic	Low CHS 0–2 (*n* = 364, 37.4%)		Medium CHS 3–4 (*n* = 474, 48.8%)		High CHS 5–8 (*n* = 134, 13.8%)		
	Mean	SD	Mean	SD	Mean	SD	*p* a
Age (yrs)	62.4	12.6	62.2	13.0	60.7	13.0	0.40
Education (yrs)	14.2	2.7	14.8	2.7	15.4	2.6	<0.001
**Cardiovascular health metrics**							
Smoking (cigarettes smoked/wk in past year)	16.7	47.4	6.1	31.2	1.0	12.1	<0.001
BMI (kg/m^2^)	31.7	6.2	28.5	5.2	25.6	4.6	<0.001
Physical activity, (MET-hours/wk)	11.1	19.4	24.6	29.4	31.7	31.9	<0.001
Total cholesterol, (mg/dL)	211.2	40.2	198.9	39.1	182.9	31.7	<0.001
SBP (mm Hg)	139.4	18.8	128.5	21.4	115.9	18.9	<0.001
DBP (mm Hg)	73.8	9.63	69.5	9.59	64.8	9.39	<0.001
Fasting blood glucose (mg/dL)	110.1	36.9	93.7	19.9	87.3	9.0	<0.001
RFS (0–23)	9.8	3.0	10.9	2.9	12.0	2.9	<0.001
Non-RFS (0–15)	4.1	1.7	3.6	1.6	2.8	1.8	<0.001
**Additional health variables**							
HDL-cholesterol (mg/dL)	51.6	14.5	53.9	15.6	57.2	15.4	0.001
LDL-cholesterol (mg/dL)	127.2	34.2	120.2	33.1	106.3	25.9	<0.001
Depression (CES-D score)^b^	8.6	7.4	7.0	6.6	6.8	5.9	0.001
tHcy (µmol/L)	10.2	4.0	10.0	3.5	9.1	2.8	0.008
Waist circumference (cm)	101.2	14.9	93.4	13.9	86.2	13.0	<0.001
Triglycerides (mg/dL)	182.2	153.3	126.4	70.7	95.6	50.0	<0.001
C-reactive protein (mg/dL)	0.52	0.53	0.41	0.48	0.26	0.26	<0.001
Alcohol intake (g/wk)	30.1	68.7	40.7	74.8	35.5	49.1	0.09
Total food serves per day	14.9	4.5	14.8	4.5	14.7	5.2	0.81
**Categorical variables**	*n*	%	*n*	%	*n*	%	*p*
Gender							0.49
Males	141	38.7	203	42.8	55	41.0	
Females	223	61.3	271	57.2	79	59.0	
CVD^c^	58	15.9	59	12.4	22	16.4	0.27
Diabetes mellitus^d^	77	21.2	42	8.9	2	1.5	<0.001
Hypertension^e^	276	75.8	268	56.5	54	40.3	<0.001
Obesity (≥30 kg/m^2^)	183	51.5	165	35.3	21	15.7	<0.001
Antihypertensive medication	217	59.6	218	46.0	49	36.6	<0.001
Cholesterol-lowering medication	102	28.0	125	26.4	39	29.1	0.77
Diabetes mellitus medication	60	16.5	39	8.2	2	1.5	<0.001

*BMI* body mass index, *CES-D* Centre for Epidemiological Studies Depression Scale, *CHS* Cardiovascular Health Score, *CVD* cardiovascular disease, *DBP* diastolic blood pressure, *HDL* high-density lipoprotein, *hs-CRP* high sensitivity C-reactive protein, *LDL* low-density lipoprotein, *MET* metabolic equivalent, *non-RFS* non Recommended Food Score, *RFS* Recommended Food Score, *SBP* systolic blood pressure, *SD* standard deviation.

a ANOVA for continuous variables and Chi-square for categorical variables.

b For CES-D, higher score indicates greater number of depressive symptoms.

c CVD was defined as present if there was self-reported history of coronary artery disease, myocardial infarction, congestive heart.

failure, transient ischemic attack, or angina pectoris.

d Diabetes mellitus defined as fasing plasma glucose ≥126 mg/dL or treated.

e Hypertension defined as ≥140/90 mm Hg or treated.

### Cardiovascular Health Score and Cognition

Associations between the three CHS categories and mean performance levels for the cognitive composite scores, the Global Composite, the Similarities test, and the MMSE are shown in Table 2 (basic and full covariate sets). Cognitive performance levels increased in a linear fashion for seven out of eight outcomes as the number of ideal cardiovascular health metrics increased (exception was for Verbal Memory), with statistical adjustment for age, education and gender (*p*<0.05 for linear trend, for all). Participants with at least five metrics in the ideal range performed significantly better on Working Memory, Scanning and Tracking, Executive Function, and on the Global Composite, compared to those with two or fewer ideal metrics (all *p*<0.05). When the full covariate set was employed, significant associations remained between cardiovasular health and performance on the Global Composite, Working Memory, and Executive Function (all *p*<0.05). No associations were found for the quadratic trend component (*p*>0.05).

**Table 2:** Multivariate-adjusted mean and SE for cognitive outcome scores according to Cardiovascular Health Score^c^ group (*N* = 972).

Cognitive outcome measure	Covariate set^a^ ^b^	Cardiovascular Health Score (0–8)							
		Low CHS 0–2 (*n* = 364)		Medium CHS 3–4 (*n* = 474)		High CHS 5–8 (*n* = 134)		*p* overall	*p* linear trend
		Mean	SE	Mean	SE	Mean	SE		
Global Composite	Basic	−0.109	0.041	0.059^d^	0.036	0.131^d^	0.068	0.002	0.003
	Full	−0.099	0.043	0.053^d^	0.037	0.090	0.073	0.016	0.030
Visual-Spatial Memory	Basic	−0.110	0.044	0.080^d^	0.038	0.069	0.072	0.003	0.034
	Full	−0.114	0.046	0.083^d^	0.039	0.057	0.077	0.005	0.064
Verbal Memory	Basic	−0.055	0.047	0.044	0.041	0.026	0.078	0.28	0.38
	Full	−0.043	0.050	0.033	0.042	−0.003	0.084	0.51	0.68
Working Memory	Basic	−0.084	0.049	0.009	0.043	0.234^d^	0.081	0.004	0.001
	Full	−0.066	0.052	−0.009	0.044	0.182	0.088	0.06	0.017
Scanning Tracking	Basic	−0.086	0.041	0.040	0.036	0.108^d^	0.067	0.017	0.014
	Full	−0.070	0.043	0.040	0.036	0.058	0.072	0.10	0.12
Executive Function	Basic	−0.071	0.046	0.000	0.040	0.205^d^ ^e^	0.076	0.008	0.002
	Full	−0.06	0.049	−0.015	0.041	0.121	0.082	0.18	0.06
Similarities	Basic	−0.077	0.047	0.044	0.041	0.089	0.077	0.08	0.07
	Full	−0.066	0.050	0.039	0.042	0.051	0.084	0.25	0.24
MMSE	Basic	−0.098	0.049	0.057	0.043	0.110	0.081	0.024	0.029
	Full	−0.066	0.051	0.049	0.043	0.081	0.086	0.18	0.15

*BMI* body mass index, *LDL* low density lipoprotein, *MMSE* Mini-Mental State Examination.

a Based on 8 health components: smoking, BMI, physical activity, total cholesterol, blood pressure, fasting plasma glucose, 2 dietary indices: Recommended Food Score, non-Recommended Food Score.

b Basic covariate set: values are mean *z*-scores after adjustment for age, education, gender.

c Full covariate set: values are mean *z*-scores after adjustment for age, education, gender, total food serves/day, triglycerides, depression, C-reactive protein and total homocysteine.

d significantly different from the low group.

e significantly different from medium group.

Sensitivity analyses with the CHS treated as a continuous variable confirmed the linear trend observed with the categorical regression analyses. The CHS was positively associated with the same seven cognitive outcomes (*p*<0.05 for all, basic covariate set) (Table S2). With the addition of other cardiovascular and psychological variables (full covariate set), significant positive associations remained between CHS and performance on Visual-Spatial Memory, Working Memory, Scanning and Tracking, Executive Function, and the Global Composite (all *p*<0.05).

There were too few ethnic minority groups to employ ethnicity in the covariate sets, but exclusion of the ethnic minority group variable from the analyses did not alter the findings (data not shown).

### Individual Health Components and Cognition

#### Health behaviors

**Figure 1:**
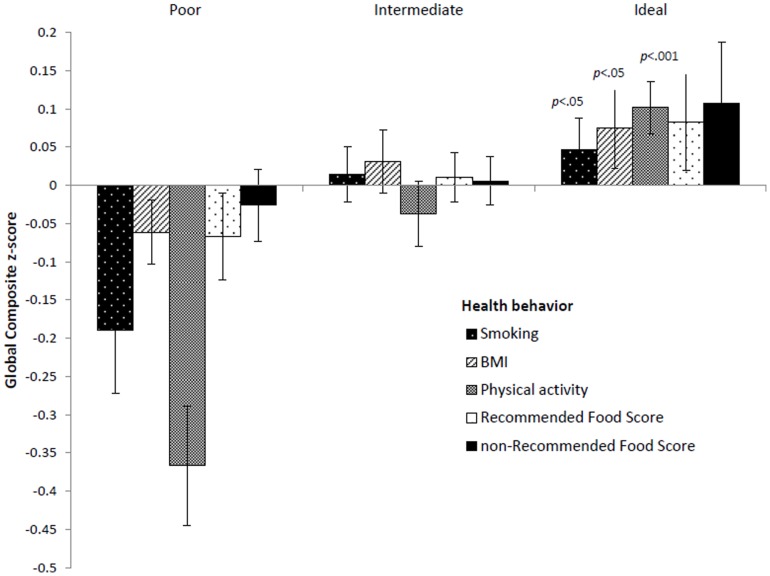
Mean Global Cognition Composite scores according to cardiovascular health level for each health behavior. Scores are mean Global Composite *z*-scores (and standard errors) for poor, intermediate and ideal health categories for smoking, BMI, physical activity, Recommended Food Score, and the non-Recommended Food Score, adjusted for age, education and gender. *p*-values are for significant linear trends.

The mean Global Composite *z*-scores for each health behavior (smoking, BMI, physical activity and diet) according to poor, intermediate and ideal levels of cardiovascular health are shown in Figure 1. The Global Composite score increased significantly as health levels increased from poor to ideal, for smoking, BMI, and physical activity (*p* for linear trend <0.05, for all). This was true with adjustment for age, education, and gender. Athough not statistically significant, Global Composite scores were also the highest for those with ideal cardiovascular health levels on the two diet scores. With full adjustment for the other cardiovascular health components, positive associations remained for smoking and physical activity (data not shown).

#### Health factors

**Figure 2:**
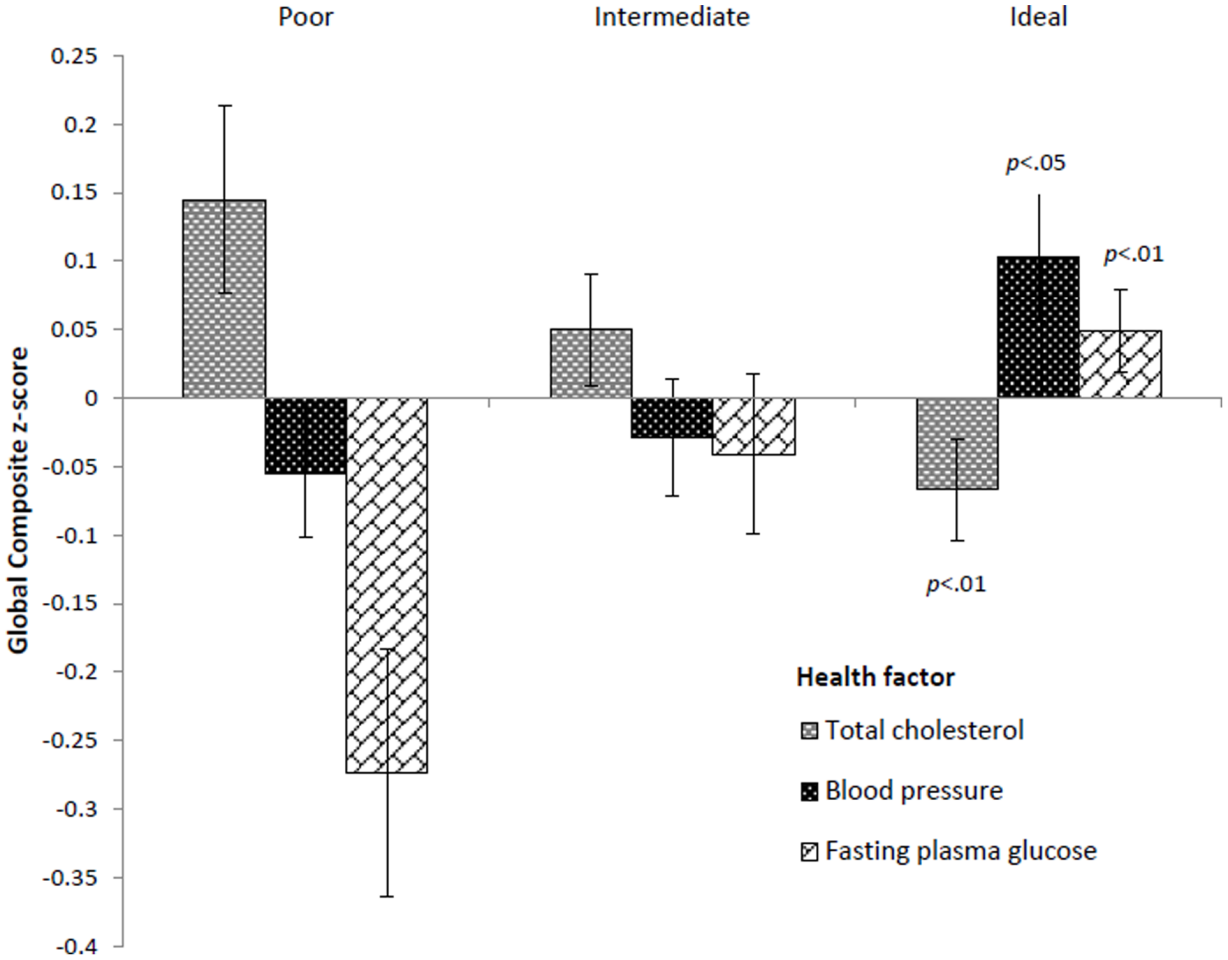
Mean Global Cognition Composite scores according to cardiovascular health level for each health factor. Scores are mean Global Composite *z*-scores (and standard errors) for poor, intermediate and ideal health categories for total cholesterol, blood pressure, and fasting plasma glucose, adjusted for age, education and gender. *p*-values are for significant linear trends.

Mean Global Composite *z*-scores for each health factor (total cholesterol, blood pressure, and fasting plasma glucose) according to cardiovascular health level are shown in Figure 2. There was a positive association between the Global Composite score and health level for BP and fasting blood glucose (*p* for linear trend <0.05, for both). With full adjustment, positive associations remained for fasting plasma glucose (data not shown). Total cholesterol displayed a significant inverse relationship with cognitive performance (*p* for linear trend <0.05, for both covariate sets).

### A Posteriori Examination of CHS x Age Interactions

As a final check on the possibility of age interactions with CHS in relation to the cognitive variables, *a posteriori* analysis was done comparing younger and older age groups, with the former defined as individuals under 60 years of age and the latter aged 60 years or more. Despite using age groups that maximized the number of subjects in each group, there were fewer subjects in the older (*n* = 429) than the younger (*n* = 543) group. Performance on the Global Composite increased in a linear fashion as the number of ideal cardiovascular health metrics increased for both age groups. For those aged under 60 years, the multivariate adjusted mean *z*-score and 95% CI, for those with the lowest CHS (0–2) was 0.273 (95% CI = 0.169–0.379), compared with a mean of 0.585 (95% CI = 0.419–0.750) for those with a CHS between 5 and 8 (*p* = 0.006), with statistical adjustment for age, education and gender. In general, effect sizes and differences among means were smaller for the older group but the pattern of results for the younger versus older participants was the same. For those aged 60 years or more, those with a CHS of 0–2 had a mean Global Composite *z*-score of −0.594 (95% CI −0.719–−0.469), compared to those in the highest CHS category with a mean score of −0.474 (95% CI = −0.691–−0.257), *p* = 0.056.

## Discussion

In this community-based study of adults, a greater number of ideal cardiovascular health components (a higher CHS) was positively associated with neropsychological test performance on a range of cognitive domains. Visual-Spatial Memory, Working Memory, Scanning and Tracking, Executive Function and Global composite scores were associated with higher CHS, and this was true with adjustment for demographic variables, cardiovascular risk factors and psychological variables. The fact that multiple cognitive functions were associated with CHS suggests that high levels of health have a generalized association with cognitive function.

Less than 1% of the sample (0.6%) achieved ideal health on seven or eight metrics. This is consistent with other US data, with studies reporting similarly very low prevalences, ranging from 0–0.1% [43]–[45] to 1.2% [46]. In 2007 to 2008, virtually no children in the USA meet all seven criteria for ideal cardiovascular health, while most adults (60%) had 2, 3, or 4 criteria at ideal levels [45]. In the present study, 71% had between 2 and 4 components at ideal levels. What is of greatest concern is the national data indicating that the prevalence of ideal cardiovascular health actually decreased from 2.0% in 1988–1994 to 1.2% in 2005–2010 [46].

The present results extend and refine findings from the CARDIA study [23]. In the CARDIA study, multiple metrics of ideal cardiovascular health in young adults (18 to 30 years) were positively associated with better performance on tests of psychomotor speed, executive function and verbal domains up to 25 years later. More specifically, CARDIA participants who had at least five ideal cardiovascular health metrics over the study period had better cognitive performance on all three tests. In the present investigation, participants with at least five metrics in the ideal range performed better on Working Memory, Scanning and Tracking, Executive Function, and on the Global Composite, as compared to those with two or fewer ideal metrics.

In a broader framework these results extend work done with the Framingham Heart Study in which the Framingham Stroke Risk Profile, a combination of weighted cardiovascular risk factors were related to lower cognitive performance on several individual measures [14]. However, the Framingham Scale includes risk factors, but does not include other health behaviors such as diet. It emphasizes risk factors that predict stroke, while the AHA-based scale used in this study emphasizes the attainment of ideal levels of risk factors. In comparison with the CAIDE dementia risk scale, the AHA scale also includes diet, and the physical activity criteria are more stringent; ideal health for physical activity is the attainment of at least 150 minutes of moderate activity per week [20], compared with 40–60 minutes per week in the CAIDE risk scale [17]. Both the Framingham stroke and CVD risk scores and the CAIDE dementia score have been shown to predict cognitive decline in late middle age [15].

The importance of the inclusion of behavioral factors in addition to traditional CVD risk factors was demonstrated in this study. Positive linear associations between each of the health behaviors (non-smoking, engaging in physical activity, maintaining a normal BMI, consuming more recommended foods) and Global cognitive performance were observed (although statistical significance was not reached for diet). Blood glucose levels and BP were inversely related to the Global Composite score. Reviews of the literature indicate that BP, diabetes, smoking, hypertenson and obesity are major risk factors for cognitive performance [7]. SBP, hypertension, diabetes, cigarette smoking status, history of CVD, atrial fibrilation, and left ventricular hypertrophy were all associated with lower levels of cognitive performance for multiple cognitive domains in the Framingham Heart Study [1], [47]–[49] as was the overall risk factor score. Thus our findings with respect to the individual risk factors are consistent with the literature.

As obesity places an individual at greater risk for type 2 diabetes, hypertension and elevated blood lipid concentrations [50], and may exacerbate cognitive decline in those with poorer cardiometabolic health [51], [52], it is a key component of cardiovascular health. The high levels of obesity (38% with BMI≥30 kg/m^2^) and physical inactivity in the present study sample are alarming. At least 150 minutes per week of moderate intensity physical activity was not achieved by 44% of participants. Time spent sedentary has been associated with poorer cardiometabolic biomarkers [53], metabolic syndrome [54], and CVD [55]. Increasing cardiovascular fitness may result in improved vascular health and increase brain tissue perfusion, which may result in cognitive gains [56], [57]. The evidence for the adverse effects of smoking on health are overwhelming; the toxic products added to the bloodstream from cigarette smoke contribute to the development of atherosclerosis, the main underlying pathophysiologic process of CVD and its clinical manifestations, as well as inducing a systemic inflammatory response [58], [59].

In the present study, participants with ideal levels for total cholesterol had significantly lower scores on global cognition measures (*p*<0.001 for both covariate sets). While this may seem counter to the expectation that ideal levels would result in better cognitive performance, positive associations between total serum cholesterol and cognition have been previously reported [60], [61]. The adverse effects of low serum cholesterol on cognitive performance have biologic plausibility as neuronal cells require cholesterol for normal metabolic processes, and cholesterol is important for brain development and maintenance of brain function [62].

Limitations include the use of self-reported measures of diet and physical activity, measured at one point in time, which may not accurately reflect long-term patterns. The study is cross-sectional, which prevents any conclusions regarding causality to be made. An acknowledged limitation is the lack of a caloric measure of total energy intake, which was unable to be obtained from the questionnaire used. This was therefore estimated using total daily serves of all foods from the dietary questionnaire. Finally, the limited number of older participants in the present study limits the generalizability of the findings.

There are a number of strengths and unique aspects to this study. Firstly, we utilized a community-based sample with extensive data on cardiovascular risk factors providing the metrics needed to calculate the CHS. Secondly, we examined multiple cognitive domains (composite scores), each indexed by multiple tests of cognitive ability as determined by previous factor analyses [26]. Therefore, the present study is the first to have assessed the combined effect of multiple cardiovascular risk factors on global cognition and multiple cognitive domains taken from an extensive neuropsychological test battery. Thirdly, a different measure of a healthful diet than that defined by the AHA and used in CARDIA [23] was implented. The AHA diet metric is based on achieving recommended intakes of fruit and vegetables, fish, high-fiber whole grains, and minimising intakes of sodium and sugar-sweetened beverages [20]. Given the growing evidence for the role that diet may play in cognitive function [2], [3], [63], the healthy diet score as defined by the AHA was expanded so as to include intake of a greater number of foods in line with the current national guidelines [64] and to obtain a greater indication of dietary variety. Fourthly, a wide age range including middle-aged and elderly participants was used. However there were no interactions between age (continously distributed) and cognitive performance, which suggests the findings extend to older groups beyond the young to middle-aged adults examined in CARDIA. Further inspection and analysis of results for persons over and under 60 years of age were highly similar in terms of differences among means for all composite scores with some reduction in magnitude of effect for the older individuals and a marginal *p* value for the older groups. There are several possible reasons for this finding. Sample size was smaller in the older group (429 versus 543), we excluded older persons with dementia, acute stroke and on dialysis, and the MSLS sample exhibites a very low level of cardiovascular events and CVD despite the presence of risk factors. Moreover, this finding is consistent with previous cross-sectional research in which associations between hypertension and cognition were stronger in young and early middle age than in older individuals [65], [66] and may be due to the fact that persons who do less well do not survive into old age or self-select out of cross-sectional studies. In the context of this literature and the characteristics of our sample, absence of age interactions with respect to relations between CHS and cognitive performance make logical sense. These relations are best examined in the context of longitudinal designs. Future studies focusing on specific age groups are needed. Finally, following recommendations for future research in a previous paper [67] we examined relations between the individual cardiovascular metics and cognitive outcomes.

### Conclusions

A higher CHS, indexed by risk factors, lifestyle factors and health measures, was associated with performance on multiple cognitive domains and global cognitive function. With control for other cardiovascular and psychological factors, this association remained significant for the Global Composite,Visual-Spatial Memory, Working Memory, Scanning and Tracking, and Executive Function composite scores. Ideal health scores for smoking, BMI, physical activity, BP, and fasting blood glucose were all positively associated with the Global Composite score. As has been previously reported [43]–[46], relatively few study participants met the AHA definition of ideal cardiovascular health in the present study.

### Implications

The personal and financial burden of cognitive decline and dementia, in terms of medical costs, quality of life and productive-years lost, are significant [25]. The AHA 2020 goals of improving health behaviors and reducing preventable cardiovascular risk factors gains support from the present study. However, by virute of the design we are unable to separate casue and effect relations. Those with better cognitive performance may engage in better health behaviors. However, it is clear from our observational data that better cardiovascular health and improved cognition are associated. Longitudinal analyses and eventally clinical trials will be necessary to clarify the direction of these associations.

## Supporting Information

Table S1 ***BMI*** **body mass index,** ***DBP*** **diastolic blood pressure;** ***non-RFS*** **non-Recommended Food Score,** ***RFS*** **Recommended Food Score,** ***SBP*** **systolic blood pressure.** ^a^ As defined by the American Heart Association, for adults >20 years of age, with exception of diet (RFS and non-RFS) [17]. ^b^ RFS out of 23, with higher scores indicating a higher consumption of recommended foods to increase. ^c^ non-RFS out of 15, with higher scores indicating a higher consumption of foods recommended to reduce.
(DOCX)

Table S2 *p<.05, **p<.01. ^a^ Basic covariate set: b is adjusted for age, education, gender. ^b^ Full covariate set: b is adjusted for age, education, gender, total food serves/day, triglycerides, depression, C-reactive protein and total homocysteine.
(DOCX)
