# Supplementary material for: Cardiovascular Health and Cognitive Function: The Maine-Syracuse Longitudinal Study
Source: PLoS One. 2014 Mar 3;9(3):e89317. doi: 10.1371/journal.pone.0089317 (PMC3940600; doi:10.1371/journal.pone.0089317)
Supplement: Table S1 — BMI body mass index, DBP diastolic blood pressure; non-RFS non-Recommended Food Score, RFS Recommended Food Score, SBP systolic blood pressure. a As defined by the American Heart Association, for adults >20 years of age, with exception of diet (RFS and non-RFS) [17]. b RFS out of 23, with higher scores indicating a higher consumption of recommended foods to increase. c non-RFS out of 15, with higher scores indicating a higher consumption of foods recommended to reduce. (DOCX) [file pone.0089317.s001.docx]

**Table S1.** Definitions of poor, intermediate and ideal health for each Cardiovascular Health component, and prevalence of each in the present study.

| Health metric^a^ | Poor health | | | Intermediate health | | | Ideal health | |
| --- | --- | --- | --- | --- | --- | --- | --- | --- |
|  | Definition | % |  | Definition | % |  | Definition | % |
| Smoking | Current smoker | 10.2 |  | Former smoker | 50.3 |  | Never smoked | 39.5 |
| BMI | ≥30 kg/m^2^ | 38.0 |  | 25-29.9 kg/m^2^ | 38.5 |  | <25 kg/m^2^ | 23.6 |
| Physical activity | None  (0 MET-minutes) | 10.5 |  | 1-149 min/wk moderate intensity  or 1-74 min/wk vigorous intensity  (1-499 MET-minutes) | 33.5 |  | ≥150 min/wk moderate intensity  or ≥75 min/wk vigorous intensity (≥500 MET-minutes) | 56.0 |
| Total cholesterol | ≥240 mg/dL | 13.4 |  | 200-239 mg/dL | 38.4 |  | <200 mg/dL | 48.3 |
| BP | SBP ≥140 or  DBP ≥90 mm Hg | 31.8 |  | SBP 120-139 or  DBP 80-89 mm Hg | 35.1 |  | <120/<80 mm Hg | 33.1 |
| Fasting plasma glucose | ≥126 mg/dL | 7.9 |  | 100-125 mg/dL | 19.0 |  | <100 mg/dL | 73.0 |
| RFS^b^ | RFS 0-8 | 21.7 |  | RFS 9-13 | 61.6 |  | RFS 14-23 | 16.7 |
| non-RFS^c^ | non-RFS 5-15 | 28.8 |  | non-RFS 2-4 | 61.3 |  | Non-RFS 0-1 | 9.9 |
