# Supplementary material for: Cardiovascular Health and Cognitive Function: The Maine-Syracuse Longitudinal Study
Source: PLoS One. 2014 Mar 3;9(3):e89317. doi: 10.1371/journal.pone.0089317 (PMC3940600; doi:10.1371/journal.pone.0089317)
Supplement: Table S2 — *p<.05, **p<.01. a Basic covariate set: b is adjusted for age, education, gender. b Full covariate set: b is adjusted for age, education, gender, total food serves/day, triglycerides, depression, C-reactive protein and total homocysteine. (DOCX) [file pone.0089317.s002.docx]

**Table S2.** Raw regression coefficients (b) and 95% confidence intervals (CI) expressing the relationship between Cardiovascular Health Score (0-8) and cognitive outcome variables (*z*-scores).

| Cognitive outcome | Basic covariate set | | | | Full covariate set | | | |
| --- | --- | --- | --- | --- | --- | --- | --- | --- |
|  | b | 95% CI | R^2^ |  | | b | 95% CI | R^2^ |
| Global Composite | 0.064** | 0.028, 0.100 | 0.380 |  | | 0.050* | 0.011, 0.090 | 0.397 |
| Visual-Spatial Memory | 0.049* | 0.011, 0.087 | 0.310 |  | | 0.046* | 0.004, 0.088 | 0.324 |
| Verbal Memory | 0.031 | -0.010, 0.072 | 0.192 |  | | 0.020 | -0.025, 0.066 | 0.209 |
| Working Memory | 0.072** | 0.029, 0.115 | 0.363 |  | | 0.051* | 0.004, 0.099 | 0.136 |
| Scanning Tracking | 0.052** | 0.016, 0.087 | 0.402 |  | | 0.040* | 0.001, 0.078 | 0.425 |
| Executive Function | 0.066** | 0.026, 0.106 | 0.242 |  | | 0.048* | 0.004, 0.092 | 0.249 |
| Similarities | 0.049* | 0.008, 0.090 | 0.200 |  | | 0.038 | -0.007, 0.084 | 0.216 |
| MMSE | 0.052* | 0.009, 0.095 | 0.132 |  | | 0.030 | -0.016, 0.077 | 0.158 |
